# *Fusarium* spp. in Human Disease: Exploring the Boundaries between Commensalism and Pathogenesis

**DOI:** 10.3390/life13071440

**Published:** 2023-06-26

**Authors:** Anca Cighir, Anca Delia Mare, Florina Vultur, Teodora Cighir, Suzana Doina Pop, Karin Horvath, Adrian Man

**Affiliations:** 1 Department of Microbiology, George Emil Palade University of Medicine, Pharmacy, Sciences and Technology of Târgu Mures, 38 Gheorghe Marinescu Street, 540139 Târgu Mures, Romania; anca.cighir@umfst.ro (A.C.);; 2 Doctoral School of Medicine and Pharmacy, George Emil Palade University of Medicine, Pharmacy, Sciences and Technology of Târgu Mures, 38 Gheorghe Marinescu Street, 540139 Târgu Mures, Romania; 3 Department of Medical Laboratory, Mureș Clinical County Hospital, 1 Gheorghe Marinescu Street, 540103 Târgu Mures, Romania; 4 Department of Ophthalmology, George Emil Palade University of Medicine, Pharmacy, Sciences and Technology of Târgu Mures, 38 Gheorghe Marinescu Street, 540139 Târgu Mures, Romania; 5 Ophthalmology Clinic, Mureș Clinical County Hospital, 1 Gheorghe Marinescu Street, 540103 Târgu Mures, Romania

**Keywords:** *Fusarium* spp., infection, colonization, molecular diagnostic

## Abstract

*Fusarium* is a large fungal genus that is widely distributed in the environment, mostly known for its plant pathogenicity. Rarely, it is involved in human pathology, where the type of infection caused is highly dependent upon the portal of entry and the immune status of the host. The study at hand aims to summarize routine methods used in diagnosing such infections as well as more advanced molecular diagnostic methods, techniques that can play a huge role in differentiating between colonization and infection when trying to decide the therapeutic outcome. Consequently, to further support our findings, two different strains (one isolated from corneal scrapings and one isolated from purulent discharge) were analyzed in a clinical context and thoroughly tested using classical and modern diagnostic methods: identification by macroscopical and microscopical examinations of the culture and mass spectrometry, completed by molecular methods such as PCR for trichothecene and ERIC-PCR for genetic fingerprinting. Isolation of a clinically relevant *Fusarium* spp. from a sample still remains a diagnostic challenge for both the clinician and the microbiologist, because differentiating between colonization and infection is very strenuous, but can make a difference in the treatment that is administered to the patient.

## 1. Introduction

*Fusarium* is a large genus that exhibits global distribution and can be found everywhere in the surroundings, including the soil and marine or river environments, being characteristic of the northern temperate regions [1,2]. It is part of the *Ascomycota* phylum and comprises more than 300 species, but only about 70 of them have been studied more in depth [1,3]. It is believed that there are approximately ten complexes that are involved in human pathology, including *Fusarium solani*, *Fusarium oxysporum*, *Fusarium fujikuroi*, *Fusarium incarnatum-equiseti*, *Fusarium dimerum*, etc. [2].

Aside from its implications in human pathology, *Fusarium* spp. is an important genus due to its pathogenicity against plants, as it causes a complex to manage disease and is a very common threat to maize, wheat and other grain cultures [2,4]. Furthermore, it exhibits toxic metabolites called mycotoxins, which can contaminate a very wide range of food products from asparagus, figs, soybean, vegetables, and spices to medicinal plants [3,5].

In humans, *Fusarium* spp. causes a variety of infections, which are highly dependent upon the portal of entry and the immune status of the host [6]: in immunocompetent people, it is the most common etiological agent of superficial infections such as keratitis and onychomycosis, but it can appear in other organs causing infections such as peritonitis in patients receiving dialysis [7,8], thrombophlebitis [9], arthritis [10,11], osteomyelitis [12], endophthalmitis [13,14], fungemia [15], sinusitis [16] and pneumonia [17]; in severely immunocompromised patients, locally invasive or disseminated infections are more frequent and are usually associated with positive blood cultures [6,18]. The species that are most commonly involved in human infection are *Fusarium solani*, followed by *Fusarium oxysporum*, *Fusarium verticillioides* and *Fusarium moniliforme* [18].

In non-immunosuppressed patients, the usual risk factors include traumatic injuries or foreign bodies in a previously colonized patient. This usually involves keratitis in people who wear contact lenses and onychomycosis in people who walk barefoot in agricultural fields [4].

Severe cases of fusariosis have been found in patients who have a weaker immune system due to risk factors including prolonged neutropenia, corticosteroid treatment, diabetes [19], solid organ transplantation or T-cell immunodeficiency and in stem cell transplant patients who develop graft-versus-host disease [18,19]. Symptoms of infection usually include persistent fever that does not respond to any antibiotic or antifungal agents [4]. Disseminated fusariosis has a death rate of 100% and it occurs mostly in hematological malignancies or in patients with severe and extensive burns [4,20].

Aside from their direct effect on the human organism, *Fusarium* spp. strains also manifest their pathogenicity through their secondary metabolites. Mycotoxins can have two types of effect: direct effects due to the consumption of mycotoxin-contaminated food, which can lead to an altered host-immune response to different infectious agents or indirect effects by altering the virulence of various infectious pathogens, leading to modifications in the toxicity and invasiveness of the microorganisms in various organs or immune cells [21].

Diagnosing fusariosis can be challenging, but these infections can be detected through a rigorous clinical examination combined with laboratory findings in a patient who presents several risk factors [18].

Currently, diagnostic methods are based on culture, microscopy and histopathological examinations. The sample taken for analysis differs based on the site of the infection: in pulmonary infections—sputum, bronchoalveolar lavage, tissue biopsy; in skin infections—skin biopsy; in disseminated infections—blood cultures [6].

If possible, direct microscopical examinations from the pathological product should be performed, as they can offer a quick diagnosis and can help the clinician initiate the antifungal treatment faster than waiting for the culture results [6]. Furthermore, this could aid in differentiating between infection and a contamination of the culture media, as the presence of hyphae in the direct microscopy confirms the presence of the pathogen in the sample and excludes all other external contamination possibilities [6].

The next step is inoculating the sample on the appropriate culture media, followed by incubation. *Fusarium* spp. grows on most media without cycloheximide such as Sabouraud dextrose agar or potato dextrose agar [6]. When inoculated on potato dextrose agar, *Fusarium* spp. produces fast-growing white, lavender, pink, salmon or grey colonies with a cottony surface [4,22]. In microscopic examinations, the filaments are hyaline and septated, and typically branch at acute or right angles. Characteristic of *Fusarium* spp. are the macroconidia and the microconidia. Macroconidia are hyaline, multicellular clusters of cells that have the shape of a banana or gondola and are supported by a foot cell at the base. Microconidia are hyaline, unicellular and have an ovoid to cylindrical shape. If microconidia are present, it is important to determine the shape, number of cells and mode of cell formation as this can aid in a preliminary species differentiation [1,4].

Polymerase chain reaction (PCR) can also be used to differentiate between different species of *Fusarium* spp., directly from the pathological product or by extracting DNA from culture [6,23].

The aim of this study is to present the common and complementary molecular diagnostic methods useful in *Fusarium* spp. infections, which aside from the clinical findings may bring important information in the differentiation between colonization and infection when trying to reach a final diagnostic. The main purpose of this paper is to highlight the importance of several microbiological methods that should be implemented in the current practice and that could aid the clinician in diagnosing such infections.

The remainder of this paper continues by outlining the considered materials and methods in Section 2, where the main laboratory techniques used are described, followed by Section 3, which highlights the results obtained by implementing the aforementioned methods. Finally, Section 4 reviews and compares the available literature with our findings and highlights the similarities and contradictions. Lastly, Section 5 draws the conclusions of our study and highlights the findings previously presented in the paper.

## 2. Materials and Methods

This section will present the materials used to achieve the final purpose of this study. This part will be divided into subcategories, each one of those subsections representing one step in the diagnostic protocol that was followed, starting from the collection and cultivation of the pathological samples and up to more complex diagnostic molecular methods.

### 2.1. Clinical Isolates of Fusarium spp. and Methods of Identification to the Level of Genus

**Figure 1:**
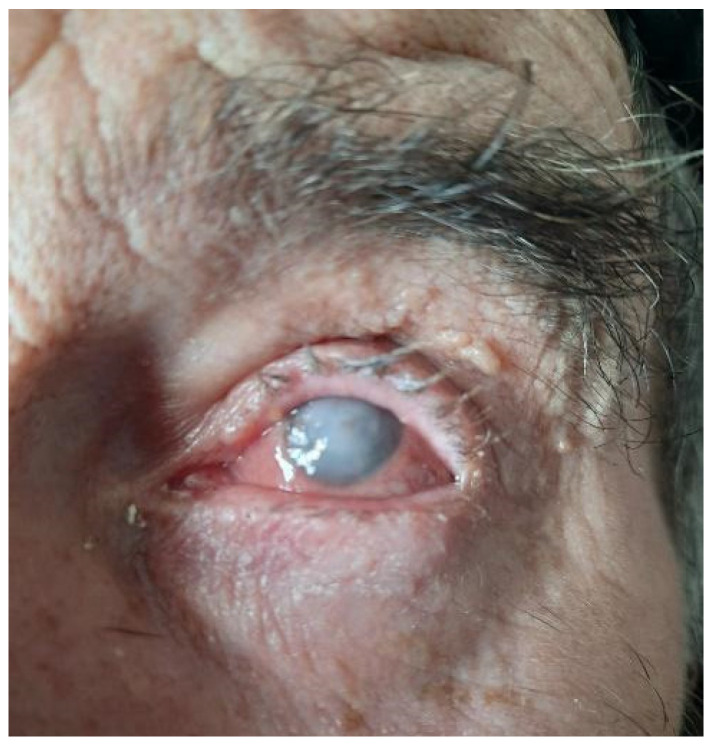
Macroscopical examination of the eye, showing the left eye lesion with white deposits.

The first *Fusarium* strain was isolated from a 70-year-old male patient with no past medical history, originally from the countryside. The patient presented himself to the ophthalmology clinic complaining of pain, photophobia, loss of visual acuity and redness of his left eye (Figure 1). The symptoms began approximately one week before; therefore, he was admitted to the hospital for further investigations and treatment. Several paraclinical tests were performed, including computer-tomography scans, left eye ocular echography (showing subtle vitreous opacities of the left eye), ocular biopsy, and bacteriological and mycological cultures from corneal scrapings for microbiological diagnosis.

The second strain was isolated from an 87-year-old male, who was admitted to the cardiology department for congestive heart failure and several associated diseases: aortic, tricuspid and mitral valve deficiencies, tetraparesis due to an ischemic stroke in the past, bronchopneumonia with respiratory insufficiency and severe atherosclerosis of the lower limbs (grade IV). All his comorbidities were associated with a lower limb (right hallux) ulcer with signs of infection (purulent secretion), from which microbiological samples were taken, followed by tissue sampling for histopathological examinations and debridement to clean up the wound to further enhance the healing process.

The pathological products from the two patients, which included corneal scrapings and purulent discharge, respectively, were sent to the microbiological laboratory for testing. As the etiology of the infection was unknown and could only be suspected based on the aspect of the lesions, the samples were inoculated on several culture media for bacteria and fungi, according to the internal laboratory protocols: blood agar, lactose agar, chocolate agar, mannitol salt agar and Sabouraud dextrose agar, and then further incubated at 35 °C for 18–24 h for bacteria and 32 °C for up to 5 days for fungi, in a normal atmosphere.

For identification, suspected fungal colonies were further isolated on Sabouraud dextrose agar and identified to the level of genus based on the aspect of the culture and by microscopical examinations using lactophenol cotton blue staining.

### 2.2. Species Identification Using MALDI-TOF MS

For identification to the level of the species, *Fusarium* spp. was isolated on Sabouraud dextrose agar and sent to a reference laboratory where supplementary tests were performed using matrix-assisted laser-desorption ionization flight time—MALDI-TOF MS (Bruker, Billerica, MA, USA). There are several MALDI-TOF MS systems available on the market for the identification of filamentous fungi, most studies being performed on Saramis [24], Vitek MS [25] or Andromas [26]; however, the most studied one in the literature is Bruker [27,28], the one that was also available in our laboratory.

For identification, the following protocol was used: spores were detached from the culture using a sterile inoculating loop and added to a sterile tube containing liquid Sabouraud dextrose agar, then added to a rotator for 24 h to encourage the formation of a fungal pellet at the bottom of the tube.

The supernatant was discharged, and the pellet was transferred to the MALDI target using a sterile pipette and overlaid with 1 μL of HCCA matrix and analyzed with MALDI-TOF.

The results were available in less than 5 min from the beginning of the analysis and were then interpreted according to the Bruker recommendations, using the Bruker commercial database [27]. Identification was based on the level of similarity between an unknown specimen and a reference sample, indicated by a log score. For bacterial cultures, the log score was interpreted as follows: higher than 2.3 indicates a “highly probable species identification”, a score between 2 and 2.299 indicates a “definite genus identification but only a probable species identification”, a score between 1.7 and 1.999 indicates a “probable genus identification” and a score under 1.7 indicates an “unreliable identification”. For filamentous fungi, there are several publications showing different perspectives on this matter, but the mean lower thresholds range from 1.7 to 2.0 [27,29,30,31].

### 2.3. Molecular Testing of Fusarium spp.

Polymerase chain reaction (PCR) was used to further analyze particular traits of the two isolated strains. The identification of virulence factors followed the presence of *Tri13* gene (involved in the production of trichothecene), which can likewise be used to confirm genus identification of *Fusarium* spp. Due to the fact that both patients were admitted to the hospital in a 2-week time slot, the relationship between the two isolates was further studied by enterobacterial repetitive intergenic consensus PCR (ERIC-PCR), a genetic fingerprinting method that is commonly used for the enterobacteriaceae family, but can also be applied to *Fusarium* spp. [32]. The principle of this method is the production of several amplicons with variable molecular sizes and specific electrophoresis patterns.

#### 2.3.1. Fungal DNA Extraction

Fungal DNA was extracted using a Zymo Quick-DNA™ fungal/bacterial miniprep kit, according to the kit protocol, with slight modifications. For this, the *Fusarium* isolates were subcultured on Sabouraud dextrose agar and incubated at 32 °C for 2 weeks to ensure the proper sporulation of the strains. The media with mature colonies were frozen at −80 °C for 45 min, and 50 mg of mycelia was scraped from the surface of the culture media, using a sterile slide, in a tube containing 200 μL of DN-ase free water and beating beads. The DNA was extracted and purified following the manufacturer’s instructions. The quantity and quality of the obtained DNA were assessed using an Eppendorf D30 Biophotometer, showing concentrations of 5.4 ng/μL, and 44.9 ng/μL DNA, respectively.

#### 2.3.2. Polymerase Chain Reaction

Detection of *Tri13* gene was performed in a mix containing 12.5 μL DreamTaq Green PCR Master Mix (Thermo Fisher Scientific, Waltham, MA, USA), 0.5 μL of each forward TRI13P1 (5′-CTCSACCGCATCGAAGASTCTC-3′) and reverse TRI13P2 (5′-GAASGTCGCARGACCTTGTTTC-3′) primers, 2 μL of fungal DNA and 9.5 μL water, to reach a final reaction volume of 25 μL. The PCR protocol consisted of: 4 min initial denaturation at 94 °C, 35 amplification cycles (94 °C for 40 s, annealing at 57.5 °C for 40 s, elongation at 72 °C for 40 s) followed by final elongation at 72 °C for 10 min.

ERIC-PCR was performed in a mix containing 12.5 μL DreamTaq Green PCR Master Mix (Thermo Fisher Scientific, Waltham, MA, USA), 0.5 μL of each forward ERIC1 (5′-ATGTAAGCTCCTGGGGATTCAC-3′) and reverse ERIC2 (3′-AAGTAAGTGACTGGGGTGAGCG-5′) primers (Thermo Fisher Scientific, Waltham, MA, USA), 2 μL of fungal DNA and 9.5 μL water, to reach a final reaction volume of 25 μL. The PCR protocol was adapted from Godoy et al. [32], as follows: 5 min initial denaturation at 95 °C, 30 amplification cycles (94 °C for 30 s, annealing at 52 °C for 1 min, elongation at 72 °C for 2 min) followed by final elongation at 72 °C for 8 min.

The PCR products were resolved by gel electrophoresis, in 1% agarose gel for the detection of the *Tri13* gene and 2% agarose gel for ERIC-PCR (TopVision Agarose, Thermo Fisher Scientific, Waltham, MA, USA) containing GelRed (Biotium Inc., Fremont, CA, USA), in Tris-Borate-EDTA buffer, for 1 h at 100 V for *Tri13* gene detection and 2 h at 80 V for ERIC-PCR. A molecular ladder (GeneRuler 1 kb, Thermo Fisher Scientific, Waltham, MA, USA) was loaded in the first and last lane of both gels. The image of the final results was captured using MiniBIS Pro (Bio-Imaging Systems, Neve Yamin, Israel).

The ERIC-PCR dendrogram was generated using GelJ Software (UPGMA method, with the band matching tolerance set at 1).

## 3. Results

**Figure 2:**
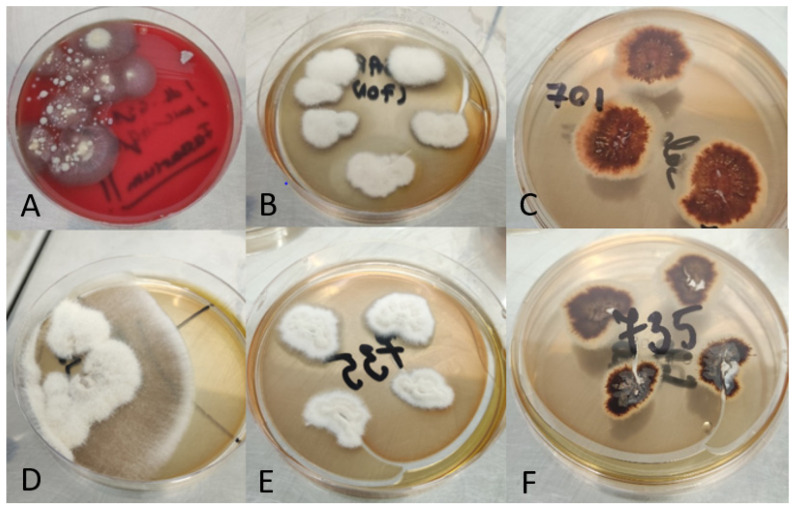
*Fusarium* spp. growth on different culture media. First sample: (**A**) Blood agar (direct culture); (**B**) Sabouraud dextrose agar averse (isolation); (**C**) Sabouraud dextrose agar reverse (isolation); Second sample: (**D**) Sabouraud dextrose agar (direct culture); (**E**) Sabouraud dextrose agar averse (isolation); (**F**) Sabouraud dextrose agar reverse (isolation).

For the first sample, because white deposits were present at eye level when we performed a physical examination of the patient, a fungal infection was taken into consideration. Therefore, even though there was no bacterial growth on the usual culture media after 48 h, it was decided to incubate them for a few more days, in case any fastidious microorganisms were present in the sample. After 5 days of incubation, white, fluffy colonies with a cottony surface and yellow-brown reverse started to grow on all culture media, with the highest colony count being seen on blood-agar (Figure 2A).

In the case of the second sample, because it was taken from a patient with arteriopathy and necrosis, the etiology of the infection was uncertain and, therefore, it was inoculated on all the common culture media. After 18–20 h of incubation, a mixed bacterial culture was present, consisting of *Morganella morganii* and *Enterococcus* spp. as the main bacterial pathogens. The Sabouraud dextrose agar media was further incubated, as white, fluffy slow-growing colonies (dark-brown reverse) started to grow, suggestive of fungal growth (Figure 2D).

The fungal colonies were further isolated on Sabouraud dextrose agar and incubated at 32 °C for up to 5 days, to obtain a pure culture. The reverse of the colonies was brown in the case of the first isolate (Figure 2C), and darker for the second isolate (Figure 2F), which suggested the implication of different *Fusarium* strains in the two patients.

**Figure 3:**
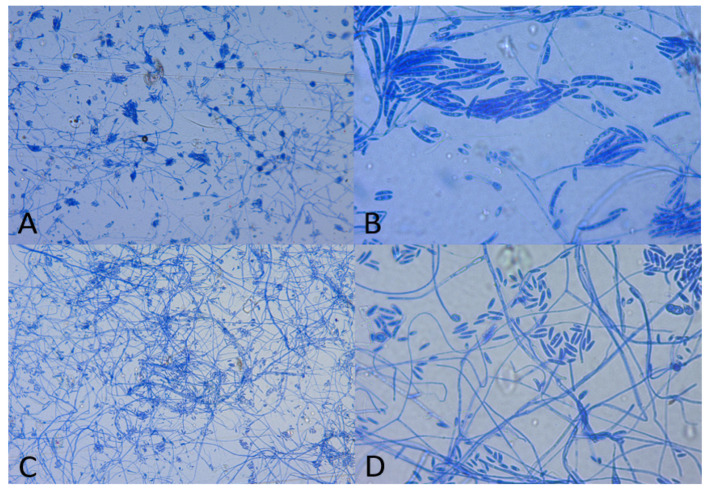
Microscopical examination using lactophenol cotton blue staining ((**A**,**B**)—sample 1; (**C**,**D**)—sample 2): (**A**,**C**)—10x objective; (**B**,**D**)—40x objective; in all pictures, characteristic banana-shaped macroconidia and microconidia can be seen.

Microscopical examination was performed from the colonies isolated on Sabouraud dextrose agar, using lactophenol cotton blue staining to facilitate identification to the level of the genus. The presence of banana-shaped macroconidia was noticed in both specimens (Figure 3). Both the macroscopical aspect of the culture and the microscopical characteristics were highly suggestive of *Fusarium* spp.

**Figure 4:**
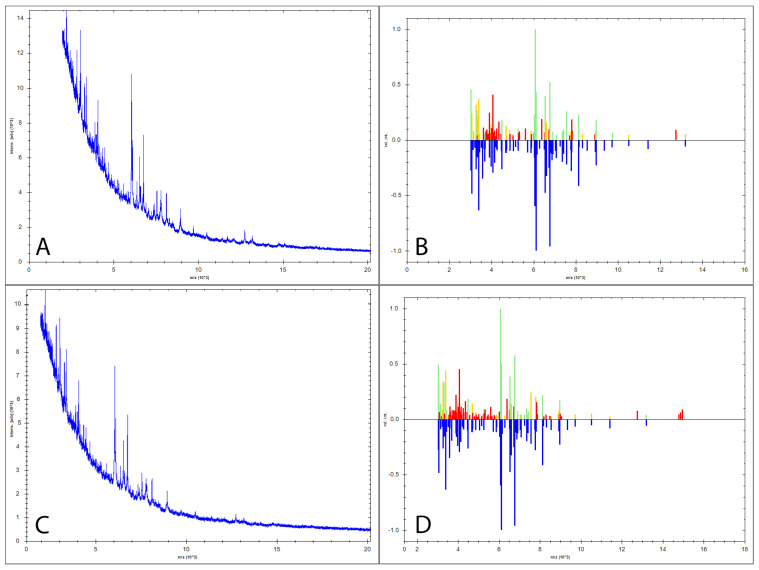
MALDI-TOF MS mass spectra graph; (**A**,**B**)—sample number 1; (**C**,**D**)—sample number 2; Images (**A**,**C**) represent the spectra detected by MALDI-TOF MS for both samples while (**B**,**D**) represent the comparison between the standard spectra from the database (above the 0.0 middle line) and the identified spectra (below the 0.0 line); *Fusarium solani* was identified based on the similarity between the sample spectra and the standard spectra, offering the log values of above 2 for both samples.

Moreover, MALDI-TOF MS further identified both fungus as *Fusarium solani*, with an identification index of 2.19 for the first strain and 2.05 for the second strain (a log score above 2 is assigned the label “high confidence identification”) (Figure 4) [33].

**Figure 5:**
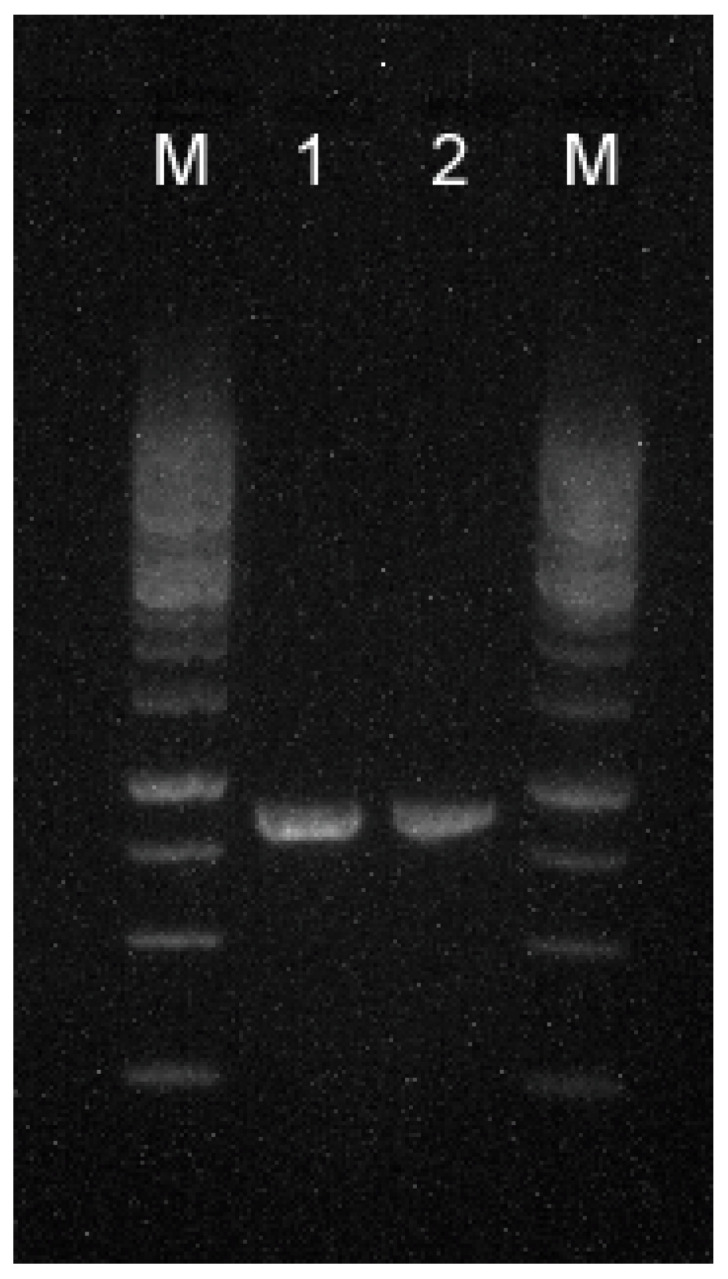
The 1% agarose gel electrophoresis wells, in order, represent: M—1 kb molecular ladder; 1—*Tri13* gene PCR amplification of the first strain; 2—*Tri13* gene PCR amplification of the second strain; fragments of about 800 bp were amplified for both strains, proving their ability to produce trichothecenes.

**Figure 6:**
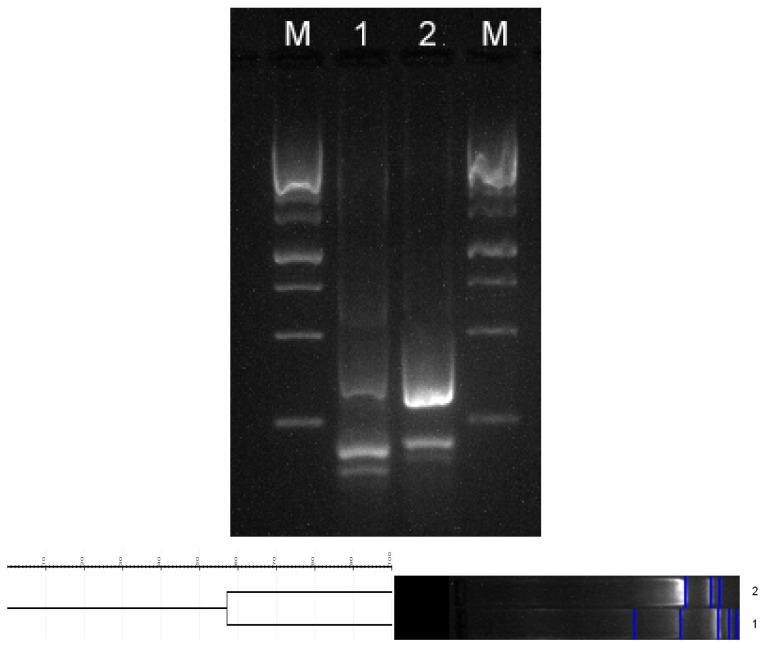
The top image shows the 2% agarose gel electrophoresis wells, which in order, represent: M—1 kb molecular ladder; 1—ERIC-PCR for the first strain; 2—ERIC-PCR for the second strain; ERIC-PCR showed different migration patterns, thus proving the genetic variability of the strains, even though they showed similarity during identification (lanes 1 and 2). The bottom image shows the dendrogram that compares the similarity between the two strains (57% similarity).

Because the fungi were isolated within a close timeframe, and because they presented morphological similarity during species identification, molecular characteristics were also evaluated for the presence of the *Tri13* gene and the genetic similarity. Both methods are cheap, fast, and easy to implement when there is a challenge in the clinical evaluation of patients. PCR for Tri13 amplified fragments of identical size of approximately 700-bp for both isolates (Figure 5—lanes 1 and 2) proved their similar ability to produce trichothecenes. ERIC-PCR proved some differences between the two fungal isolates (Figure 6—lanes 1 and 2): the first strain produced five variable-sized amplicons (~150–500 bp, with a more intense band at ~150 bp); the second strain produced three amplicons in the same molecular range, but in slightly displaced positions, and a more intense band at ~300 bp. This confirms that the two isolates belong to the same species, but are not related, presenting a similarity of 57% according to the dendrogram.

Both cases had a favorable evolution under treatment.

In the first case, once the infection with *Fusarium* spp. was confirmed, appropriate treatment was administered. The patient received subconjunctival injections with voriconazole, associated with atropine and dexamethasone. His evolution was towards healing of the lesion, but unfortunately it was associated with loss of eyesight. After one week of treatment, he went home where he continued his treatment with voriconazole drops and tablets for 5 more days.

In the second case, because bacteria were considered the main pathogens, no specific antifungal treatment was administered. The lesion was thoroughly surgically debrided and topical antibiotic creams were applied in order to treat the infection. The evolution of this case was also favorable towards healing.

## 4. Discussion

Generally, the incidence of fusariosis is variable depending upon the region, being influenced by climatic conditions. In the case of eye infections with *Fusarium* spp., the highest incidence can be noted in tropical countries, while in temperate regions the incidence is as low as 10% and highly dependent on other factors such as socioeconomic status, climate and involvement in agriculture [34]. For skin infections with *Fusarium* spp., the incidence is also higher in tropical countries, and has been slowly but surely increasing in recent years [35].

In Romania, as presented in one of our previous studies [36], the incidence of fusariosis was surprisingly high. In a timeslot of 10 years, from a total number of 68 cases that were positive for filamentous fungi, 8 cases were infections with *Fusarium* spp. (11.76%), most of them coming from purulent discharge secretions; one exception was the isolate addressed in this study, which originated from the corneal scrapings of a patient with endophthalmitis. Furthermore, the number of cases of infection with this fungus was much higher during the COVID-19 pandemic (six positive isolates) than before the pandemic when only two cases of fusariosis were found. Both cases presented in this paper were isolated during the COVID-19 pandemic, a couple of weeks apart.

*Fusarium* spp. are widely distributed in the environment, including water and the soil, and they are significant plant pathogens. Taking this into consideration, it is expected that *Fusarium* spp. will eventually colonize the external surfaces of humans, with or without initializing infection; additionally, it is highly probable that this fungus will be isolated as associated microflora in infections, but its clinical involvement is hard to prove. Nevertheless, even in immunocompetent patients, it can cause limited, localized lesions. However, in immunocompromised patients (e.g., burn patients, hematological patients, etc.) the infections are usually invasive or disseminated [37]. The most common infections caused by this fungus are onychomycosis, and keratitis [37], which was also present in one of our cases.

Even though there is a variety of species involved in mycotic keratitis, *Fusarium solani* is the most common etiological agent [38]. In *Fusarium* spp. keratitis, clinical examinations are not of great help in diagnosis as symptoms are quite similar to those of other bacterial or fungal infections. Therefore, in order to ensure proper diagnosis, a culture of corneal scrapings or a tissue biopsy is required [18]. In our case, the proper diagnosis (even just to the level of the genus) was of great aid, as it turned out the patient was receiving treatment with an antifungal drug that had no effect on *Fusarium* spp (Fluconazole). After the diagnostic was made, the antifungal drug was changed, and the patient’s status evolved towards healing. He managed to keep the eye, but unfortunately he completely lost his eyesight on the affected side.

Keratomycosis usually appears in the presence of different risk factors such as corneal trauma (commonly with vegetative matter) or, more recently, due to the use of contact lenses. Other factors include: farming, long-term use of antibiotics or topical steroids, diabetes, HIV infection, previous ocular surgery, or any affections of the eye [34]. What is interesting in our case is the fact that the patient had no previous lesions or known risk factors for developing fungal endophthalmitis, besides the fact that he worked in agriculture in a rural area. Moreover, the *Fusarium solani* strain was isolated from a lesion in a site where the microflora is normally well controlled; this, together with the lack of any bacterial pathogens, makes us conclude that this fungus was the etiological agent of the infection, and not a colonizer.

*Fusarium* skin infections have a wide variety of symptoms, directly related to the part they affect. In the case of onychomycosis, they usually present as a dense, white discoloration of the nail bed. If the skin is affected and it causes tinea pedis, a maceration aspect is present, usually between the fingers of the lower limb, which can easily be confused with dermatophyte infections; therefore, only microbiological examinations can establish a final diagnosis [39]. Alternatively, at the skin level, disseminations from a generalized fusariosis can appear, or superinfections of the ulcerated areas of the skin such as diabetic or arterial ulcers [39].

In our case, the patient presented significant risk factors (aortic, tricuspid and mitral valve deficiencies, tetraparesis due to an ischemic stroke in the past, bronchopneumonia with respiratory insufficiency and grade IV atherosclerosis of the lower limbs) that could make us think of an ulcer superinfection with *Fusarium* spp., due to vascular insufficiency, but only histopathological examinations could truly reveal if it was an actual infection or just colonization. As the histopathologic examination showed no fungal involvement, it can be concluded that in this particular case, *Fusarium solani* was only a superficial colonizer. Furthermore, after the patient had received local treatment consisting of wound debridement and local antibiotic creams, the evolution of the ulcer was towards crust formation and healing.

The topic of colonization versus infection is widely discussed nowadays, as one involves a mandatory antifungal emergency treatment and the other can be left untreated or, in the case of skin infections, treated through a thorough debridement of the wound. But how can we differentiate between the two?

Due to the fact that *Fusarium* spp. can contaminate laboratory materials, for accurate diagnostic several criteria should be taken into consideration: for the strain to be isolated from multiple culture media or to identify multiple colonies of the same fungus on a culture media and for the pathological sample to be relevant to the fungus isolated (e.g., isolation from an immunocompromised patient is highly relevant and should always be taken into consideration when a diagnostic is performed, while isolation from the skin scrapings of an immunocompetent individual should raise the question of a contamination and make the microbiologist take into consideration the idea of asking the clinician for another sample) [18]. In high-risk patients, the presence of hyphae and yeast-like structures in the same tissue specimen is highly suggestive of a fusariosis [6].

Furthermore, if a strain is isolated from a symptomatic patient and from a sterile site in a patient with elevated markers of infection in the blood, it is a suggestive sign of infection. If the strain is isolated from a superficial skin wound, from an atypical site or from a patient who is immunocompetent, the chances of this being considered an infection are low, and we therefore lean more towards considering this a colonization.

In our case, the first patient had characteristic symptoms of endophthalmitis, as well as slight neutrophilia. Because the *Fusarium solani* strain was isolated from a sterile corneal scraping, this could lead us to believe that he had an active infection. In the second patient, the lack of any symptoms (besides the skin lesion and slightly increased inflammatory markers), the polymicrobial findings from the lesion, as well as the favorable evolution of the wound with only minor treatment support the diagnostic of a fungal colonization rather than that of fungal etiology of an infection. Moreover, the sample type (purulent discharge), taken from a superficial part of the wound, presents an increased risk of being colonized with fungi.

Currently in our hospital, microbiological diagnostic is limited to identification to the level of the genus based on standard methods of identification: cultivation on Sabouraud dextrose agar and macroscopical examination followed by microscopical examination using lactophenol cotton blue. Unfortunately, these methods are only reliable up to a certain degree, because many *Fusarium* species look similar in culture. Furthermore, the media and growth conditions used influence the growth rate and colony pigmentation, as well as the formation of the structures that aid in differentiating between species and the dimensions of those structures [40]. Even if this is time-consuming, as cultures may take up to 2 weeks to grow, cultivation and microscopy still remain valid diagnostic methods, especially when more accurate alternatives are not available.

MALDI-TOF is one of the fastest and most accurate ways of identification, not just for bacteria, but also for fungi, but unfortunately, it is not widely available, especially in smaller laboratories [41]. Furthermore, PCR-based identification methods were also explored. The literature was researched to find some suitable primers that were easy to use and reliable and could constitute a possible future diagnostic method. Several primers for species identification were found [42,43,44,45], but we finally settled on a primer that could be universally used and would detect all species of *Fusarium* spp., being able to tell us if the fungus was present in the sample or not.

Besides the classical diagnostic methods in fungal infections, molecular methods bring valuable information on patient management, participating in the clinical and epidemiological management. The identification of virulence factors of the etiological agents involved in infections is part of the findings that have to be checked whenever possible. For example, in *Fusarium* species, an important virulence factor is the capacity to produce mycotoxins. Evaluation of the *Tri13* gene was described in many studies as an important finding in *Fusarium* spp. [44], as it can show exactly what type of mycotoxins are produced, based on their molecular length following PCR. The *Tri13* primers are designed to identify different conserved regions of both functional and non-functional *Tri13* alleles in both nivalenol (NIV)- and deoxynivalenol (DON)-producing isolates [44]. If a fragment of approximately 799-bp is amplified, the strain is a DON-producing strain. If a 1075-bp fragment is amplified, it is a NIV-producing strain. Since in our study amplicons of about 800-bp were generated, it can be concluded that both isolates are DON-producing. The secreted mycotoxins may have played a role in the tissue damage.

Studies have shown that, besides the effects it has on human health when ingested together with food products, DON can affect the immune system of the host by causing cytotoxicity and an increase in cell apoptosis [46,47]. Furthermore, it can have dose-dependent immunosuppressive or immunostimulant effects, which can also contribute towards the virulence of the fungi, as well as aid in the virulence of the bacterial strains involved in skin infections [46]. In mice experiments, DON proved to be a main factor that contributed to the reduction in the white blood cells population as well as altering antibody production [21]. More specifically, IgM and delayed-type hypersensitivity responses to bacterial infections are majorly suppressed [21].

Very little information is currently available regarding the effects DON-producing *Fusarium* strains have on ocular infections. Anutarapongpan et al. [48] conducted a study of 50 different strains of *Fusarium* spp. involved in fungal keratitis, without finding any DON- or NIV-producing strains. Instead, they found fumonisin (FUM)-producing strains, and showed that the levels of these mycotoxins were correlated to a faster progression of the disease towards penetrating keratoplasty and a worsening of visual acuity. Schrecker et al. [49] described in their study a case of *Fusarium* keratitis in a patient without any risk factors, similar to our case. The authors assume the reason why the fungus was able to infect the perfectly healthy eye was due to the production of appropriate virulence factors such as enzymes and the capacity to produce cytotoxins, all of these components being able to be modulated by DON production, as mentioned before.

ERIC-PCR is a technique mostly used for genotyping bacteria, but studies suggest that it is a good discrimination method for fungi as well [32]. In our case, the results could clearly differentiate between the two strains, even though they were identified by MALDI-TOF MS as belonging to the same species. The method has its limitations, especially regarding fungal isolates, and it yields the best results when combined with PCR-restriction fragment length polymorphism (PCR-RFLP) [32].

## 5. Conclusions

Fusariosis is slowly becoming a “common” rare fungal infection because its incidence is steadily but surely increasing as more and more cases arise worldwide. Therefore, it is becoming imperiously necessary to be able to diagnose and treat this fungal pathogen.

Identifying the fungus is also a key point in diagnosis. Today, laboratory diagnostics are undergoing many changes as old laboratory techniques are gradually replaced by faster, more accurate methods of identification based on molecular techniques. Nevertheless, culturing the pathogen and microscopical examinations still remains a key element in small laboratories where resources are limited.

When talking about species identification, MALDI-TOF is the fastest and most accurate way of diagnosing these types of infections. Other molecular methods such as PCR are becoming available, but up to this point, with the available information, it unfortunately still is a method that is not widely available, especially in smaller laboratories. Nevertheless, if specific multiplex PCR primers can be developed for species identification, they could become a viable and accurate diagnostic method in the future. On the other hand, with the currently available resources, PCR can be used as a fast and valid method for genre identification of different virulence factors of those strains, to study the production of different mycotoxins and even for genotyping of the fungal strains.

The biggest challenge in diagnosis remains, to this day, differentiating between colonization and infection. As with other pathogens, when trying to establish a diagnosis, several important factors should be taken into consideration: the symptoms of the patient and if they are at risk of acquiring the infection; whether the pathogen has a clinical significance once isolated from the specimen and whether it correlates with the paraclinical examinations (e.g., inflammatory markers) and radiological examinations. Once all these factors have been taken into account, then and only then can the physician affirm with certainty which of the two situations they are dealing with.

## Data Availability

Data are available upon request from the authors.
